# Vitamins, Minerals and Phytonutrients as Modulators of Canine Immune Function: A Literature Review

**DOI:** 10.3390/vetsci11120655

**Published:** 2024-12-16

**Authors:** Carolina Barroso, António J. M. Fonseca, Ana R. J. Cabrita

**Affiliations:** REQUIMTE, LAQV, ICBAS, School of Medicine and Biomedical Sciences, University of Porto, Rua Jorge Viterbo Ferreira 228, 4050-313 Porto, Portugal; cibarroso@icbas.up.pt (C.B.); ajfonseca@icbas.up.pt (A.J.M.F.)

**Keywords:** dogs, immune responses, minerals, nutrition, phytonutrients, vitamins

## Abstract

Trends in the pet food industry are driven by the humanization of pets, favoring the inclusion of functional ingredients or supplements that promote animal health. Several commercial diets claim to include supplements with benefits for dogs’ immune function, but in vivo evidence that supports their efficacy remains limited. This literature review aimed to better understand the current knowledge on the effects of vitamins, minerals and phytonutrients on dogs’ immune function. A total of 27 peer-reviewed articles were identified in PubMed and Web of Science databases. Although vitamin supplementation is often claimed to support immune function, only two studies promoting slight benefits of vitamins C and E were found. The limited research on minerals suggests that organic sources promote a better immune response. Studies evaluating the inclusion of different phytonutrients show that these compounds might exert immunomodulatory and anti-inflammatory effects. Despite the increased popularity of commercial diets claimed to support the immune response of dogs, further research is needed in order to substantiate their effects. This knowledge will contribute to the development of effective diets to enhance immune health in dogs.

## Simple Summary

As pets are increasingly seen as family members, there is a growing interest in incorporating functional supplements into pet food to improve animal health. Several commercial dog diets claim to support normal immune system function, but scientific evidence is limited. This literature review summarized scientific studies on the in vivo effects of vitamins, minerals and plant-based compounds (phytonutrients) on the immune responses of canines. A total of 27 studies were analyzed from scientific databases. While vitamin supplements are frequently marketed for immune support, only two studies demonstrated modest benefits of vitamins C and E. Research on minerals suggests that organic forms may more effectively enhance immune function than inorganic ones. Furthermore, phytonutrients may contribute to immune system regulation and reduction of inflammation. Despite the growing interest in diets targeting immune health, further research is necessary to corroborate these effects in order to develop biologically effective functional diets for dogs.

## 1. Introduction

Nowadays, dogs have become an invaluable companion animal while also assuming a multifaceted role within our society, including serving as guides, therapists and law enforcement officers [1]. According to the European Pet Food Industry Federation (FEDIAF), the number of dogs in Europe surpassed 106 million in 2022, compared to approximately 93 million in 2021 [2,3]. This increase in the number of dogs and other pets increases the demand for foods, but also raises questions about the sustainability and environmental impact of the pet food industry [4,5]. Additionally, pet owners are increasingly seeking diets that provide not only the essential nutrients for their animals but that also present functional properties to enhance their dog’s health [6]. Nutritionally balanced diets supplemented with functional additives have long been recognized to benefit the overall health and disease resistance of production animals [7,8,9,10], as well as receiving considerable attention in companion animals such as dogs [11,12]. A recent survey of 762 pet owners from Canada (24%), France (28%), the United Kingdom (21%) and the United States of America (28%) concluded that 55% of owners look for ingredients and supplements that benefit gut health and digestion, 47% look for food that supports the immune system, followed by food that strengthens joints and bones (46%) and supports heart and cardiovascular health (40%) [13]. Regarding the important role of immunity in animal health and the well-known diet effects, interest in nutritional immunology is an emerging field of study.

Micronutrients, such as vitamins and minerals, are involved in regulating and shaping an immune response. However, most studies on modulation of immune responses through micronutrients have been performed with rodents and humans, with limited knowledge on companion animals [14]. Similarly, phytonutrients, a group of compounds that are naturally present in plants and considered not essential for dogs, may confer health benefits, namely, on immune responses. However, a recent review summarized the roles of plant-based ingredients and phytonutrients in canine nutrition and health and concluded that, despite the existence of an association between intake of plant foods or phytonutrients and canine health, this theme is in its infancy and warrants further investigation [15].

Therefore, despite the availability in the market of commercial diets claimed to enhance immune function and reduce inflammation, scientific evidence of the in vivo effects of vitamins, minerals and phytonutrients on the immune responses of dogs is scarce. The present review aimed to fill this knowledge gap, contributing both to identifying the need for future research and to helping the pet food industry, in particular, companies dedicated to market additives and supplements, fostering informed supplementation strategies to induce favorable health benefits.

## 2. Literature Search and Data Selection

**Figure 1:**
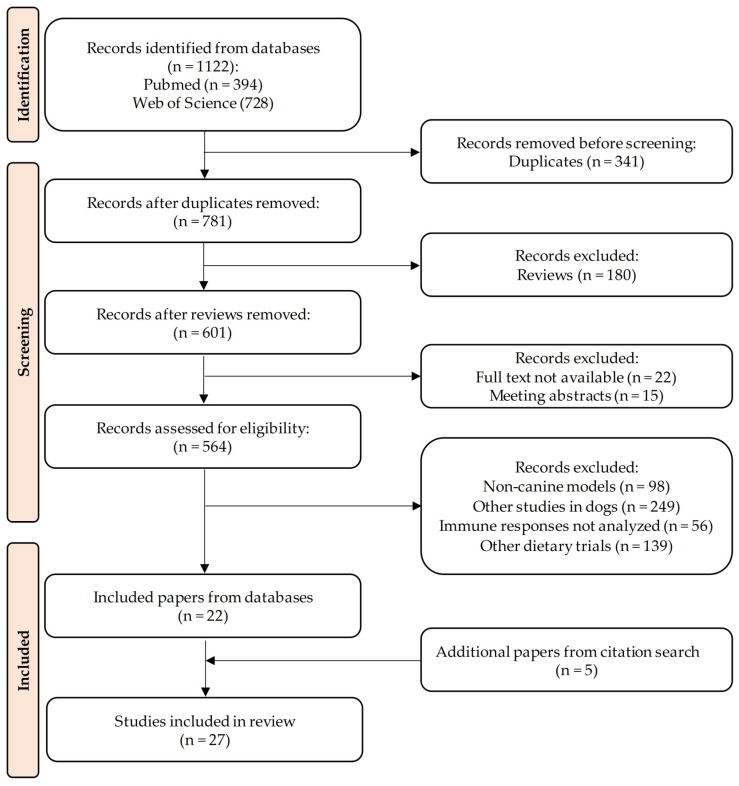
Flow diagram for the literature review. Reviews also include book chapters, editorials and opinion articles. Other studies in dogs comprise case reports, in vitro trials and in vivo studies not related to nutrition. Other dietary trials include the ones that analyzed complete foods or more than one ingredient/supplement, as well as other functional ingredients (polyunsaturated fatty acids, probiotics, prebiotics or yeast-derived ingredients).

Two electronic databases (PubMed and Web of Science) were searched for articles published in English, using the following terms in the title and abstract: (Canine OR Dogs OR Dog) AND (Dietary OR Dietary supplementation OR Ingredient OR Nutrition OR Foods) AND (Immune modulation OR Immunological OR Immune OR Immune function OR Immune response OR Inflammatory OR Immunity OR Cytokines OR Immune system OR Immunoglobulins OR Immune cells OR Phagocytic activity OR Pro-inflammatory OR Anti-inflammatory OR Innate immunity OR Acquired immunity OR Lymphocyte OR Monocyte OR Macrophage OR Neutrophil OR Granulocyte OR Immunology). All articles (n = 1122) were retrieved from databases in February 2024, inserted into an EndNote library (EndNote^TM^ 21.2, Clarivate, Philadelphia, PA, USA) and all duplicates were then removed (n = 341). The resultant list of studies (n = 781) was assessed for inclusion and 22 studies were selected. Additional studies (n = 5) cited in previous relevant articles and that met the criteria for inclusion were also added to the list of articles included in the review (n = 27; Figure 1).

The selected articles were the studies that analyzed the in vivo effects of the dietary supplementation of minerals, vitamins or phytonutrients on circulating cytokines or antibodies, fecal immunoglobulin (Ig) A, cellular or tissular expression of immune-related genes, transcriptomic analysis, immune cell phenotyping, cellular responses (lymphocyte proliferation, phagocytosis, respiratory burst, cell cytokine production) or delayed type hypersensitivity (DTH) responses of dogs. The articles that only assessed the effects of these supplements on hematology, serum biochemistry or on the markers of inflammation C-reactive protein (CRP), haptoglobin or calprotectin were excluded from the analysis.

## 3. Vitamins, Minerals and Phytonutrients as Modulators of Canine Immune Function

### 3.1. Vitamins

Vitamins are classified into fat-soluble (A, D, E, K) or water-soluble (B-complex and C) groups [16]. Most vitamins are essential for dogs and must be obtained from the diet, with the exception of vitamin C that is synthesized in the liver from D-glucose [17]. Vitamins have different roles, including in skeletal health, vision, reproduction and the immune system, however, while previous studies provide insights on the metabolism of different vitamins in companion animals, the physiological roles of these micronutrients in dogs and their potential benefits for canine health are yet to be fully understood [18,19,20,21].

**Figure 2:**
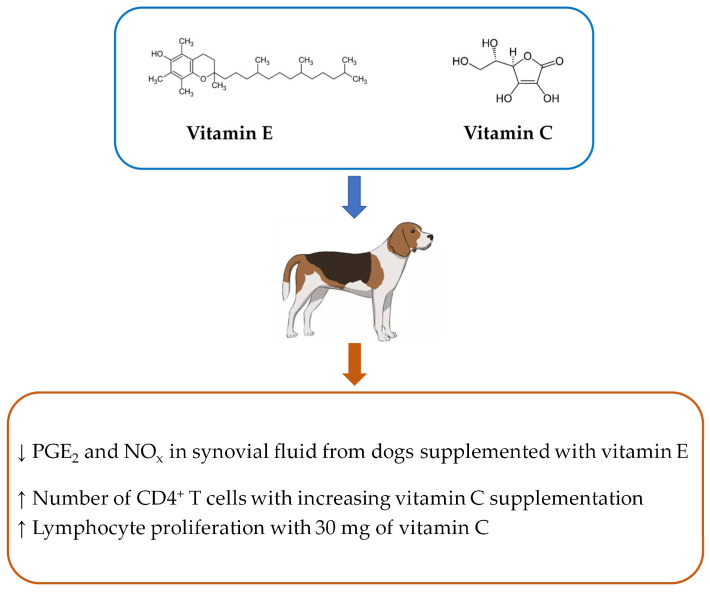
In vivo effects of vitamins E and C on dogs’ immune function. Upward arrows (↑) indicate an increase, while downward arrows (↓) indicate a decrease in the parameters analyzed.

Regarding the effects of vitamins on immune response of dogs, in vivo studies are almost inexistent and the two retrieved in this review are focused on vitamins C (ascorbic acid) and E (α-tocopherol; Figure 2; Table 1). Vitamins C and E are well-known for their potent antioxidant properties and their roles in the immune system [21,22,23]. Immune cells present a high amount of polyunsaturated fatty acids in their membranes, rendering them highly sensitive to oxidative stress, resulting in the production of lipid peroxides that can have detrimental effects [22]. Vitamins C and E are effective in protecting cells from oxidative stress by neutralizing free radicals [21,22]. The benefits of vitamin E in immune cells, particularly T cells, are linked to its role in preserving membrane integrity, influencing signal transduction and cell division. Additionally, vitamin E was also shown to inhibit the production of inflammatory mediators such as prostaglandin E2 (PGE_2_), interleukin (IL)-6 or tumor necrosis factor (TNF)-α in aged mice, particularly in response to pathogens [23]. Likewise, vitamin C can also modulate immune responses, improving neutrophil chemotaxis and phagocytosis, enhance lymphocyte proliferation and regulate the expression of pro- and anti-inflammatory cytokines [24].

Rouhma et al. [25] tested the supplementation of vitamin E in pain assessment, inflammatory markers and structural changes in the joints of adult crossbred dogs with surgically induced osteoarthritis. Animals were first subjected to the surgical procedure and then divided into two groups to receive an oral placebo solution (control) or 0.044 mL/kg of body weight (BW) of liquid vitamin E as α-tocopheryl acetate (Rovimix E-40%, 400 IU/mL/dog/day). This supplementation is ten times the daily level of vitamin E recommended by the Association of American Feed Control Officials [26], but non-toxic for dogs, as these animals can tolerate higher levels of vitamin E (1000 to 2000 IU/kg of food) without reported adverse effects [17]. The trial began one day after the surgery and lasted for 55 days. The authors observed reduced PGE_2_ and nitrogen oxide concentrations in synovial fluid and lesion scores of dogs supplemented with vitamin E, while IL-1β was below the detection limit in both groups. Although the results suggest a reduction in inflammation joint markers and a trend to improve signs of pain with a high supplementation level of vitamin E, the study presents some limitations, particularly the duration of the trial and the low number of animals used (eight dogs in the control and seven dogs in the vitamin E groups).

Hesta et al. [27] conducted a study to assess the impact of 60 mg of vitamin E (dl-α-tocopheryl acetate per dog/day) in combination with different amounts of vitamin C (0, 30 and 60 mg ascorbic acid crystalline per dog/day) on beagle dogs over three 36-day periods. The researchers observed modest effects on immunological parameters. Specifically, the number of CD4^+^ cells increased with higher vitamin C supplementation, and peripheral blood mononuclear cell (PBMC) proliferation in response to concanavalin A (ConcA), phytohemagglutinin (PHA) and pokeweed mitogen (PWM) increased with 30 mg of vitamin C but decreased with 60 mg. The authors suggested that a lower amount of vitamin C (30 mg) in combination with 60 mg of vitamin E may have stimulated the immune responses, whereas a high level (60 mg of ascorbic acid) may promote an inhibitory effect. Indeed, despite the moderate doses of vitamin C used in this study, it is known that a high level might be pro-oxidant and increase free-radical damage [28]. Although vitamins, including vitamins C and E, are well-recognized for their roles in supporting immune function, current evidence on their effects in dogs remains limited. This paucity of comprehensive research makes it difficult to draw definitive conclusions concerning the benefits of dietary vitamins on canine immunity. Further studies are essential to better understand the impact of the different vitamins on dogs, which would help the development of more efficacious and tailored dietary strategies to promote overall health and immune responses.

### 3.2. Minerals

Minerals are elements necessary in small quantities to sustain vital processes in the body and are classified into two groups: macro and trace elements [29]. Essential macro elements for dogs include calcium, phosphorus, potassium, sodium, magnesium and chlorine, while essential trace elements comprise iron, zinc, selenium, copper, manganese and iodine [17]. As both deficiency and excess of minerals can result in health problems, their absorption, utilization, storage and excretion are regulated to maintain proper body functions and prevent disorders associated with unbalanced intake [30,31].

**Figure 3:**
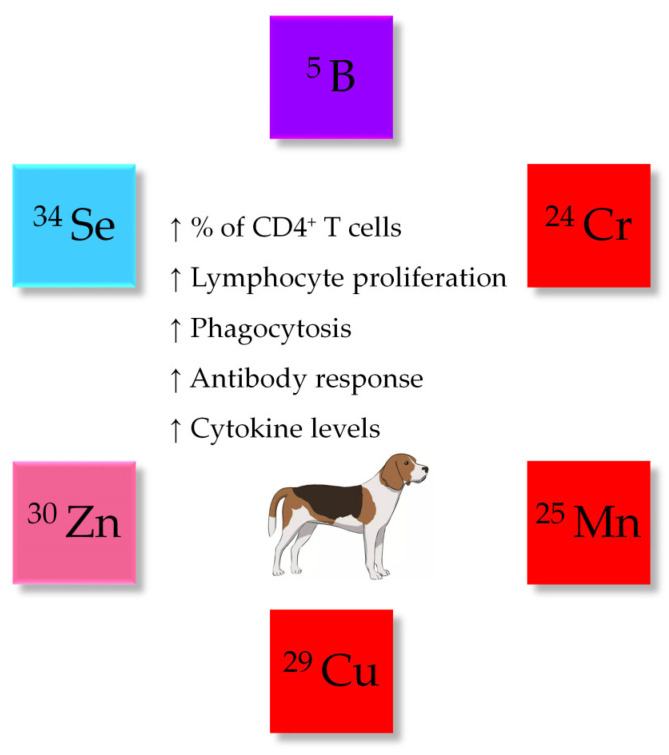
In vivo effects of trace minerals on dogs’ immune function. Purple represents metalloid trace minerals, red indicates transition metals, pink denotes post-transition trace minerals, and blue stands for reactive non-metals. Upward arrows (↑) indicate an increase in the parameters analyzed.

In vivo studies with dogs that evaluated inflammatory and immune parameters are mainly restricted to some trace elements (Figure 3; Table 2), with one study evaluating the effect of calcium fructoborate as a source of boron (CFB). This latter natural compound is found in fruits and vegetables and consists of calcium bound to fructoborate, a boron-containing carbohydrate complex [32]. Calcium, together with phosphorus and vitamin D, has a vital role in skeletal development and health while boron, not considered an essential element for humans and animals, may exert beneficial effects on growth, bone formation and immunity [33,34]. Calcium fructoborate has garnered considerable attention in human health due to its potential benefits in bone and joint health, as well as its antioxidant and anti-inflammatory properties [32,35,36]. In dogs with osteoarthritis [37], the daily ingestion of one (small dog breeds) or two (large dog breeds) capsules containing a high level of CFB (127 mg) for 28 days increased the concentration of the soluble receptor for advanced glycation end products (sRAGE) when compared to animals fed the placebo. This observation suggests a potential binding and interference with the receptor for advanced glycation end products (RAGE) that is involved in a cascade of events leading to inflammation [38], potentially leading to reduced inflammation and cartilage damage. Furthermore, dogs receiving the low (69 mg) or the high (127 mg) levels of CFB exhibited an improved ability to rise from a lying position, compared to the placebo group, but no differences were observed between dogs receiving CFB and the combination treatment (69 mg of CFB with 500 mg of glucosamine hydrochloride and 200 mg of chondroitin sulfate). Although this study suggests that the supplementation with CFB may affect the inflammatory response of dogs, effects might be dependent on the dog’s breed, size and age and the severity of osteoarthritis.

**Table 1:** Studies on effects of dietary inclusion of vitamins on immune parameters of dogs.

Vitamins				
Breed/Age	Diet	Experimental Design/Duration of the Trial	Immune Parameters	Reference
15 crossbred dogs with surgically induced osteoarthritis/1–4 years old	0 or 0.044 mL/kg BW of vitamin E, as liquid α-tocopheryl acetate (Rovimix E-40%; ~400 IU/dog/day)	Double-blinded and randomized pilot study with 8 dogs in the control group and 7 dogs in the vitamin E group/55 days	↓ PGE_2_ and NO_x_ in synovial fluid from dogs supplemented with vitamin E; IL-1β below the detection limits in both groups	[25]
7 male and 8 female beagle dogs/<2.5 and >7 years old	Vitamin E (60 mg dL-α-tocopheryl acetate) in combination with vitamin C (0, 30 and 60 mg ascorbic acid crystalline) via capsule	3 × 3 cross-over design/3 periods of 36 days	Number of CD4^+^ lymphocytes increased with increasing vitamin C supplementation; Significant time × treatment interaction on PBMCs stimulated with PWM, with an increase observed with 30 mg vitamin C and a decrease with 60 mg vitamin C; No effects on percentage of CD5^+^, CD8^+^ and CD21^+^ lymphocytes and on serum IgA and IgG concentrations	[27]

BW: Body weight; CD: Cluster of differentiation; Ig: Immunoglobulin; IL: Interleukin; NO_x_: Nitrogen oxides; PBMCs: Peripheral blood mononuclear cells; PGE_2_: Prostaglandin E2; PWM: Pokeweed mitogen. Downward arrows (↓) indicate a decrease in the parameters analyzed.

Trace elements are crucial components in several physiological and metabolic processes, also exerting antioxidant and immune functions [30,39]. Trace elements can be included in the diet in inorganic or organic forms, with inorganic forms being presented as salts, such as carbonates or sulfates, while organic trace minerals are bonded to amino acids, peptides or polysaccharides [40]. Although inorganic trace minerals are commonly used in diets for production and companion animals [40,41], their bioavailability might be compromised by their interaction with other food components, such as phytates, forming insoluble complexes [42]. In contrast, the bond of trace elements to organic compounds can prevent the formation of those insoluble complexes, increasing their absorption and bioavailability [40,43].

Studies evaluating the effects of dietary supplementation with trace elements on inflammatory and immune response of dogs are scarce and mainly focused on the comparison of inorganic and organic sources. Zinc, in particular, plays a critical role in the immune system, influencing both innate and adaptive immune responses. Research in humans and mice has demonstrated that imbalanced zinc levels disrupt the development, activation and function of B and T cells, natural killer (NK) cells, neutrophils and macrophages. Such imbalances have been associated with increased susceptibility to infections as well as a higher risk of autoimmune diseases and certain cancers [44]. Similarly, zinc supplementation was been shown to benefit the development and maintenance of the livestock immune system [45]. In canines, a recent review summarized the current understanding of zinc requirements and its biological roles [42]. However, to the best of authors’ knowledge, the relationship between dietary zinc (regardless of the source) and canine immunity remains poorly understood, with only one study published to date [46]. In this study, the authors evaluated the supplementation of 75 mg/kg of zinc sulfate or zinc proteinate, without or with 200 mg/kg of exogenous enzymes (obtained from the solid-state fermentation of *Aspergillus niger*), in diets with high phytate content, known to affect zinc bioavailability [42]. These diets were fed to beagle dogs during four periods of five weeks each, and the abundance of CD4^+^ and CD8^+^ lymphocytes was determined at the end of each experimental period. The authors observed a higher percentage of CD4^+^ cells in dogs fed zinc proteinate, regardless of enzyme supplementation, suggesting an improvement of T cell differentiation with the inclusion of an organic source of zinc. As CD4^+^ T cells are a component of the adaptive immune response, the results might suggest that the supplementation with organic zinc might have a role in the management of a diverse range of pathogenic challenges [47].

Regarding selenium sources, Shao et al. [48] evaluated the effects of dietary supplementation for 42 days with sodium selenite (0.35 mg/kg dry matter, DM) or selenohomolanthionine (SeHLan), an organic selenium biosynthesized by *Candida utilis*, at 0, 0.35, 1 or 2 mg/kg in weaned beagle puppies vaccinated for canine distemper, adenovirus type-2, parainfluenza and parvovirus (Vanguard Plus 5, Zoetis Inc., Lincoln, OR, USA) at the beginning of the feeding trial and after 21 days. In comparison to sodium selenite, 0.35 mg/kg of SeHLan increased serum selenium, IL-2 and IL-4 levels and canine parvovirus (CPV) antibody titers. Regarding the feeding level of the organic source used, dogs supplemented with 1 mg/kg of SeHLan showed the highest PBMC proliferation and serum IL-2 and IL-4 concentrations, and the group fed 2 mg/kg had the highest CPV antibody titers, while both 1 and 2 mg/kg of SeHLan promoted the highest polymorphonuclear neutrophil (PMN) phagocytosis activity. The authors concluded that a feeding level of 1 to 2 mg/kg of SeHLan is effective for improving immune function of vaccinated puppies.

In another study, Wang et al. [49] analyzed the effects of feeding beagle dogs with snacks containing 0 or 0.03% of casein phosphopeptide-selenium chelate (CPP-Se) for 30 days. Animals fed with the CPP-Se snacks showed higher lymphocyte numbers and increased serum IgM, interferon (IFN)-γ, IL-6 and IL-4 concentrations, while no effects were observed in monocyte and neutrophil numbers and serum IgG. Gene expression analysis in lymphocytes collected from dogs fed with CPP-Se revealed higher expression of *IL-4*, *IL-6*, *TNF-α*, *IFN-γ*, *CD4* and *CD8α* and a downregulation of *IL-1β*, while *IL-10* expression was lower than in controls at 10 days but was upregulated at the end of the feeding trial. The results show that CPP-Se stimulated lymphocyte immune responses by increasing their number and modulating the expression of immune-related genes. CPP-Se also influences humoral responses by increasing serum IgM but not IgG. In a second experiment with beagle dogs fed the same amount of CPP-Se for 30 days, the authors performed a peripheral blood transcriptomic and serum metabolomic analysis to further elucidate the mechanisms by which CPP-Se exerts immunomodulatory roles in dogs [50]. Transcriptomic analysis revealed several differentially expressed genes involved in immune-related pathways, such as cytokine–cytokine receptor interaction, nuclear factor kappa B (NF-κB) and T cell receptor signaling pathways. Moreover, the metabolomic analysis outcomes indicated that amino acid metabolic pathways, which are closely related to immune responses [51], exhibit significant enrichment with CPP-Se. These amino acids include tryptophan, phenylalanine, cysteine and methionine metabolic pathways.

Chromium is not included in the list of mineral supplements allowed in the European Union to be used in animal feeding, but the use of organic forms, such as chromium propionate, as a food supplement for livestock and companion animals has been approved in the USA [52]. To test the effects of this organic source in dogs, Farret et al. [53] supplemented diets with of 0 or 0.5 g/kg of KemTRACE^®^ Chromium, a commercial supplement containing 0.4% of chromium propionate (providing an additional calculated amount of 2 mg of this element per kg of food) to be fed to beagle dogs during two periods of 28 days. The authors observed higher serum CRP, IFN-γ, TNF-α and IL-10 concentrations in dogs fed the supplemented diet. Protein fractionation revealed higher levels of gamma globulins in the serum of dogs fed chromium propionate. Because gamma globulins include immunoglobulins, their increased concentration could suggest an increase in Ig levels. Thus, a feeding level of 2 mg of chromium per kg of food resulted in an increase in inflammatory markers but may also modulate humoral responses, potentially exerting a positive effect on dogs. Still, the findings of this study are insufficient to definitively conclude that chromium propionate is indeed beneficial for canines.

The study by [41] tested the replacement of inorganic with organic sources of zinc, manganese, selenium and copper (zinc oxide, manganese sulfate, sodium selenite and copper sulfate vs. minerals complexed with 2-hydroxy-4-(methylthio)butanoate, HMTBa). Both diets provided 40, 40, 0.3 and 20 mg/kg diet of zinc, manganese, selenium and copper, respectively, and were fed to adult dogs of different breeds for 30 days. On the 10th day of the experiment, animals were injected with a suspension of sheep red blood cells (SRBCs). A significant decrease in antibody titers against SRBCs was observed in dogs fed the diet containing inorganic trace minerals, from day 20 to 30, suggesting that the organic mineral source can improve the immune status of dogs.

Despite the scarcity of literature on the effects of minerals on inflammatory and immune responses of dogs, the existing studies suggest potential enhancement of immune responses with organic trace mineral supplementation compared to inorganic sources, due to the higher bioavailability of the former [42]. Further research is necessary to elucidate the long-term effects of these constituents on canine immunity.

### 3.3. Phytonutrients

Phytonutrients are naturally occurring compounds that play pivotal roles in plant defense mechanisms, providing protection against a variety of external stressors [54,55]. Despite being non-essential, these bioactive compounds show a wealth of potential benefits for human and animal health [56,57], exhibiting anti-inflammatory, antimicrobial and immunomodulatory properties [9,57,58].

Carotenoids can be divided into carotenes (with a 40-carbon structure), including α-carotene, β-carotene or lycopene, and xanthophylls, comprising lutein, zeaxanthin or astaxanthin [59]. Through oxidative cleavage, carotenoids give rise to apocarotenoids, with fewer than 40 carbons, including bixin [60]. To date, scientific in vivo studies reporting immune-enhancing benefits of carotenoids in dogs are limited to lutein, β-carotene and bixin (Table 3), with one study addressing the use of the microalga *Haematococcus pluvialis* as a source of astaxanthin [61]. Kim et al. [62] supplemented female beagle dogs with a daily inclusion level of 0, 5, 10 or 20 mg of lutein from a commercial supplement, for 12 weeks. The dogs were vaccinated for canine distemper, adenovirus type-2, parainfluenza and parvovirus (Vanguard 5^TM^, Smithkline Beacham, West Chester, PA, USA) as an antigenic challenge in weeks 13 and 15. Dietary lutein increased DTH responses to PHA and vaccine, lymphocyte proliferation response to ConcA, PHA and PWM, the percentages of CD5^+^, CD4^+^, CD8^+^ and MHCII^+^-expressing cells and plasma IgG levels after the second vaccination in a dose-dependent manner. The authors did not observe alterations in plasma IgM, CD21^+^ B cells and IL-2 production by PHA-stimulated PBMCs. Similarly, Alarça et al. [63] reported increased CD4^+^ and CD8^+^ cell counts in beagle dogs fed a diet containing 45 g/kg of active xanthophylls (76.7% of lutein and 5.2% of zeaxanthin), for 120 days, but no effects were observed in lymphocyte proliferation index. Based on these results, supplementation with lutein may enhance the overall health status of canines, potentially conferring benefits to animals with diverse pathological conditions or at various life stages [63].

**Table 2:** Studies on effects of dietary inclusion of minerals on immune parameters of dogs.

Minerals				
Breed/Age	Diet	Experimental Design/Duration of the Trial	Immune Parameters	Reference
59 client-owned dogs of different breeds with osteoarthritis/8.42 ± 0.37 years old	69 mg of calcium fructoborate (CFB; low level), 127 mg of CFB (high level) or combination of 69 mg of CFB, 500 mg of glucosamine hydrochloride (GH) and 200 mg chondroitin sulfate (CS), via capsule; 2 capsules for large dogs	Double-blinded, placebo-controlled study with 15 dogs in the placebo group, 14 dogs in the low or high CFB groups and 16 dogs in the CFB + GH + CS group/28 days	↑ sRAGE concentration between 0 and 28 days in dogs fed the high level of CFB compared to the placebo group; No effects on COMP, MMP-3, FSTL1, CTX-II, hyaluronan, Col2–3/4C, long mono, CHI3L1, IL-6 and CRP	[37]
6 male and 6 female beagle dogs/1 year old	75 mg/kg of zinc from zinc sulfate (inorganic; IZ) or zinc proteinate (organic; OZ); IZ or OZ supplemented with a commercial multienzymatic complex (phytase, protease, xylanase, β-glucanase, cellulase, amylase and pectinase) from the solid-state fermentation of *Aspergillus niger* (Synergen^®^) at a level of 200 mg/kg (IZ+ or OZ+)	4 × 4 replicated Latin square with a 2 × 2 factorial arrangement of treatments/4 periods of 5 weeks	↑ % of CD4^+^ cells of dogs fed OZ and OZ+; No effects on CRP, % of CD8^+^ cells and on CD4:CD8 ratio	[46]
30 male beagle dogs/7 weeks old	Supplementation with 0.35 mg/kg of food DM of sodium selenite (SS), 0, 0.35 mg/kg (SeHLan-L), 1 mg/kg (SeHLan-M) or 2 mg/kg (SeHLan-H) of selenohomolanthionine (SeHLan) and immunization with Vanguard Plus 5	Completely randomized design with 5 dogs per experimental group/42 days of dietary treatment, starting at the first day of vaccination (day 0); second vaccination on day 21 post-immunization (PI) as a booster dose	↑ PBMC proliferation and serum IL-6 and IL-4 in the SeHLan-M/+Vacc; ↑ PMN phagocytosis in SeHLan-M/+Vacc and SeHLan-H/+Vacc; ↑ CPV antibodies in SeHLan-H/+Vacc; Improved immune activity with SeHLan-L compared with SS/+Vacc	[48]
10 male and 10 female beagle dogs/1 year old	0 or 0.03% of casein phosphopeptide-selenium chelate (CPP-Se), provided in snacks	5 male and 5 female beagle dogs per experimental group/30 days	↑ Lymphocyte numbers, *IL-4*, *IL-6*, *TNF-α*, *IFN-γ*, *CD4* and *CD8α* expression in lymphocytes, serum IgM, IFN-γ, IL-6 and IL-4 in dogs fed CPP-Se; Downregulation of *IL-10* expression at 10 days but upregulated at 30 days in dogs fed CPP-Se; ↓ *IL-1β* expression at 30 days in dogs fed CPP-Se; No effects on monocyte and neutrophil numbers and serum IgG levels	[49]
12 adult beagle dogs/1.5 years old	0 or 0.03% of casein phosphopeptide-selenium chelate (CPP-Se), provided in snacks	6 dogs per experimental group/30 days	Immune-related pathways enriched: cytokine–cytokine receptor interaction pathway and TCR signaling pathway; Serum metabolomic analysis reveals enrichment of amino acid pathways, including tryptophan, phenylalanine, cysteine and methionine	[50]
10 adult male beagle dogs	0 or 0.5 g/kg of KemTRACE^®^ Chromium (Cr; 0.4% Cr from Cr propionate), providing an additional calculated amount of 2 mg of Cr/kg of food	10 dogs per experimental diet/two periods of 28 days, with an interval of 15 days between them	↑ Serum CRP, IFN-γ, TNF-α, IL-10 and γ-globulins in dogs fed the supplemented diet	[53]
12 male and 6 female dogs of different breeds/2 to 6 years old	Organic (OMSD; Mintrex^®^, 1 kg/t; Zn, Mn, Se, Cu bonded to HMTBa) or inorganic (IMSD; zinc oxide, manganese sulfate, sodium selenite, copper sulfate) sources that contributed with 40, 40, 0.3 and 20 mg/kg diet of Zn, Mn, Se, Cu, respectively	6 male and 3 female dogs per experimental group/30 days; injection with 1 mL of 10% suspension of sheep red blood cells (SRBCs), at 10 days of feeding trial	↓ In vivo antibody production in dogs fed IMSD, from day 20 to 30	[41]

CD: Cluster of differentiation; CHI3L1: Chitinase 3-like protein 1; Col2–3/4C, long mono: Canine collagen type II cleavage; COMP: Cartilage oligomeric matrix protein; CPV: Canine parvovirus; CRP: C-reactive protein; CTX-II: C-terminal cross-linked telopeptide type II collagen; Cu: Copper; DM: Dry matter; FSTL1: Follistatin-like protein-l; HMTBa: 2-Hydroxy-4-(methylthio)butanoate; IFN-γ: Interferon-gamma; Ig: Immunoglobulin; IL: Interleukin; MMP-3: Matrix metalloproteinase 3; Mn: Manganese; PBMCs: Peripheral blood mononuclear cells; PMN: Polymorphonuclear neutrophil; Se: Selenium; sRAGE: Soluble receptor for advanced glycation end products; TCR signaling pathway: T cell receptor signaling pathway; TNF-α: Tumor necrosis factor-alpha; Zn: zinc. Upward arrows (↑) indicate an increase, while downward arrows (↓) indicate a decrease in the parameters analyzed.

In female beagle dogs, Chew et al. [61] found that daily supplementation with 20 mg of astanxanthin from *H. pluvialis* for 16 weeks improved both cell-mediated and humoral responses of dogs by increasing DTH responses, lymphocyte proliferation response to ConcA, NK cell cytotoxicity, circulating B cells and plasma Igs. Furthermore, animals also showed an enhanced antioxidant and anti-inflammatory status, with lower levels of plasma CRP and 8-hydroxy-2′-deoxyguanosine, a marker of DNA damage. Microalgae can exert their antioxidant activities by increasing antioxidant enzymes, by preventing DNA damage and/or by scavenging free radicals [64]. According to the authors, astaxanthin is likely exerting its anti-inflammatory activity by inhibiting the action of reactive oxygen species. Furthermore, as immune cells are susceptible to oxidative damage due to the presence of polyunsaturated fatty acids in their membranes [22], the improvement of immune cell function by this carotenoid may be partially attributed to its antioxidant activity [61].

Chew et al. [65] addressed the effects of different concentrations of β-carotene (0, 2, 20 or 50 mg/day) in female beagle dogs for 8 weeks. Animals fed with 20 and 50 mg of β-carotene exhibited a heightened DTH reaction to an attenuated Vanguard 5^TM^ (Smithkline Beacham, West Chester, PA, USA) vaccine and PHA, accompanied by an increase in the percentage of CD4^+^ cells and IgG plasma levels, when compared to controls. A correlation between plasma β-carotene levels and immune responses was observed, with lower levels being associated with reduced lymphocyte proliferation and decreased IgG concentrations. Furthermore, no effects were observed in the production of IL-2 by cultured PBMCs and in plasma of dogs fed β-carotene. The lack of effects on IL-2 suggests the involvement of other cytokines in the T cell immune responsiveness that were not analyzed in this study [65]. Massimino et al. [66] analyzed the dietary supplementation of 20 or 40 mg of β-carotene in young (mean age 1.7 years old) and older dogs (mean age 10.6 years old) during two periods of 2 months each. Older dogs that were not supplemented with β-carotene showed lower percentages of T helper cells, as well as mitogen-induced lymphocyte responses, when compared to young dogs. However, treatment with 20 mg of β-carotene improved the immunological function of aged canines, resulting in a higher percentage of CD4^+^ cells and greater lymphocyte proliferation. The authors concluded that older dogs have lower immune responses, but β-carotene supplementation may influence immunocompetence of older dogs, restoring their responses [66].

The functional properties of bixin, mainly found in annatto seeds (*Bixa orellana* L.) [67], have not been thoroughly investigated. A few studies have shown its ability to exert antioxidant, anti-inflammatory, antiobesity and immunomodulatory effects in rodents and cats [68,69,70,71], while only one study has focused on the influence of bixin supplementation on dogs’ immune responses (Table 3). This study [72] tested the supplementation of 0, 5, 10 or 20 mg of bixin per day in female beagle dogs for 16 weeks and found increased DTH responses, plasma IgG levels and circulating CD21^+^ B cells, depending on the level of bixin, with no differences being observed in in vitro lymphocyte proliferation in response to PHA and ConcA. Furthermore, bixin supplementation, regardless of the dietary level administered, exhibited minor anti-inflammatory effects, since plasma CRP levels were reduced with bixin intake [72].

These findings show that different carotenoids stimulate both in vivo cell-mediated and humoral immune responses in dogs, although the precise mechanisms through which these pigments impact immune function remain underexplored.

Polyphenols constitute a large group of naturally occurring compounds from plants that are divided into flavonoids (such as anthocyanins and flavanols) and non-flavonoids (including phenolic acids and tannins) [73] and are gaining recognition for their potential as functional supplements for dogs with osteoarthritis, diabetes and insulin sensitivity or cardiovascular diseases [12,15]. A few studies have analyzed the impact of different phenolic compounds on the immune system and inflammation in dogs with obesity or diabetes (Table 3). Rahman et al. [74] reported that a high-fat diet (53% fat) supplemented with 0.25 or 0.50 g/kg of green tea polyphenols (TPs) mainly constituted by the flavanols (−)-epigallocatechin gallate (42.40 mg/g TP), (−)-epigallocatechin (13.21 mg/g), (−)-epicatechin gallate (6.18 mg/g) and (−)-epicatechin (4.51 mg/g) over a 12-week period lowered the inflammatory response of dogs with induced obesity. Similarly, Li et al. [75] observed lower circulating levels of TNF-α, IL-6 and IL-1β in obese dogs fed a high-fat diet (76% of the normal diet with 4.7% crude fat and 10% egg yolk, 10% pig oil, 2.5% cholesterol and 1.5% bile acid sodium) supplemented with 0.48, 0.96 and 1.92% TP extracts, containing 42% (−)-epigallocatechin gallate, 13% (−)-epigallocatechin, 6% (−)-epicatechin gallate and 4% (−)-epicatechin, relative to dogs fed the unsupplemented diet. The mRNA expression of these interleukins in the intestine, as well as the protein levels of Toll-like receptor 4, followed the same trend, with dogs fed high levels of polyphenols showing the lowest levels of expression. The results demonstrate that polyphenols can act as therapeutic agents for obesity and liver inflammation via cyclooxygenase-2 and inducible nitric oxide synthase inhibition and by lowering the expression of several proinflammatory cytokines [74]. Furthermore, TPs exhibit protective effects against dysbiosis caused by a high-fat diet, reduce intestinal inflammation and may attenuate the development of a chronic inflammatory condition, such as inflammatory bowel disease (IBD) [75]. During a 4-week trial, Yu et al. [76] fed young Chinese indigenous dogs with a diet containing 10% lard and 6% mulberry leaf powder (MLP), that present several bioactive peptides, including: 1-deoxynojirimycin (7.06 µg/g), gallic acid (2.51 µg/g), protocatechuic acid (9.44 µg/g), gallocatechin gallate (0.14 µg/g), catechin (0.04 µg/g), rutin (657.88 µg/g), quercetin (3.48 µg/g), chlorogenic acid (3449.12 µg/g) and naringenin (0.35 µg/g). At the end of the feeding trial, the authors failed to show significant differences in serum IgG, IgM, IL-6 and TNF-α levels among diets. However, dogs fed the control diet supplemented with 10% lard and 6% MLP presented higher serum IgA levels than controls and dogs fed 10% lard without MLP supplementation, suggesting an improvement of the dogs’ immunity with the administration of MLP.

The use of curcumin, a phenolic acid extracted from turmeric (*Curcuma longa*), has also been tested as a dietary supplement in dogs. In the study conducted by Suemanotham et al. [77], dogs with symptoms of diabetes mellitus were fed an oral turmeric extract capsule containing 250 mg of curcuminoids for 180 days. In this study, mild antioxidant effects were observed, as evidenced by the increased plasma reduced/oxidized glutathione ratio (GSH:GSSG ratio), while no effects were verified for plasma IL-6 and IL-10 levels. To the best of the authors’ knowledge, only two studies focused on the mechanisms by which curcumin influences inflammatory and immune parameters in dogs suffering from musculoskeletal disorders. Colliti et al. [78] studied the effects of curcumin on inflammatory pathways in dogs with osteoarthritis by administering 4 mg/kg twice daily, for 20 days, and performing a transcriptomic analysis of canine peripheral white blood cells before and after treatment. The results revealed a decrease in the number of genes involved in inflammatory response and in connective tissue development and function pathways after curcumin and non-steroidal anti-inflammatory drug administration. Curcumin administration was associated with an upregulation of the inhibitor of nuclear factor kappa B (IkB), known to be involved in the regulation of the NF-κB pathway [79], and downregulation of IL-18, a proinflammatory cytokine. According to the authors, curcumin could be considered as a complementary anti-inflammatory support for the treatment of osteoarthritis in dogs.

The anti-inflammatory role of curcumin was also addressed by Sgorlon et al. [80], after supplementing dogs with arthrosis with turmeric extract (6.60 mg/kg body weight of curcumin) for 60 days. These authors observed downregulation of genes including *TNF*, *NFkB1*, C-X-C motif chemokine ligand 8 (*CXCL8*) and prostaglandin-endoperoxide synthase 2 (*PTGS2*), while *SOD2* was upregulated. The same authors treated dogs actively involved in training with common bilberry (*Vaccinium myrtillus*; 0.20 mg/kg live weight of the flavonoid anthocyanidin), with animals also showing decreased *TNF*, *NFkB1*, *CXCL8* and *PTGS2* expression and increased *SOD2* [80]. The other phytonutrients analyzed in the study promoted different results. The supplementation of narrow-leaved purple coneflower (*Echinacea angustifolia*; 0.10 mg/kg live weight as the phenolic acid echinacoside, administered in healthy animals) showed decreased *TNF* and *NFkB1* but higher *CXCL8* expression, and milk thistle (*Sylibum marianum*; 1.5 mg/kg live weight as the flavonoid sylibin, in dogs with hepatopathy) promoted antioxidant responses by increasing *SOD2* [80].

A recent study addressed the anti-inflammatory effects of grape seed proanthocyanidin (GSP), a condensed tannin, on labrador retrievers with IBD [81]. Dogs were orally supplemented with 30 mg/kg BW of GSP for 28 days. Supplementation with GSP led to reduced serum TNF-α, IL-6, IL-1β and CRP concentrations when compared to dogs with IBD supplemented with a saline solution (vehicle group). Dogs from the vehicle group were then subjected to a fecal microbiota transplantation (FMT), with animals from the GSP group being used as the donors for the procedure. The results from the second experiment reflected the ones observed in the first trial, with dogs subjected to FMT showing decreased serum inflammatory markers. This anti-inflammatory profile was associated with an altered gut microbiota and microbiota-derived bile acids [81].

Gallic acid is a natural phenolic acid, found in edible plants such as tea leaf, blueberry or grape seed [82], and has been the subject of research for its potential anti-inflammatory benefits using canine models. In particular, Yang et al. [83] supplemented beagle dogs with 0, 0.02, 0.04 or 0.08% of gallic acid for 45 days. Gallic acid exerted anti-inflammatory effects by decreasing serum IL-1β and IFN-γ concentrations, regardless of the level administered, while no effects were observed on TNF-α, IL-8 and IgG. The use of gallic acid was also tested in beagle puppies with induced stress. Animals were fed with 500 mg/kg of gallic acid and subjected to transportation for 3 h [84]. The trial lasted for 14 days, with dogs being transported on day 7 after gallic acid supplementation. The effect of stress on dogs was evidenced by the alterations of serum IgG and cytokines TNF-α and IL-4, whose levels were restored to values similar to controls with gallic acid treatment. In another study, Yang et al. [85] fed beagle puppies with 2.5 g/kg of gallnut (*Galla chinensis*) tannic acid (TA), a hydrolyzable tannin, for 14 days. In the first seven days, dogs were subjected to a stressful environment (temperature of 29 °C and humidity of 96%), being then transported for 3 h to an environment with a lower temperature (23 °C) and humidity (70%). Gallnut tannic acid promoted mild anti-inflammatory effects on dogs, showing a tendency for lower serum IL-6 and higher IL-4 levels, compared to dogs subjected to stress but without supplementation, after transportation. Furthermore, dogs fed TA showed a tendency (*p* < 0.10) for higher IL-2 levels before transportation when compared to dogs with induced stress but without TA supplementation, while no significant effects of dietary treatment were observed on TNF-α levels. Both gallic and gallnut tannic acids improved the overall condition of dogs, with animals showing reduced stressed-induced gut dysbiosis, diarrheal symptoms, oxidative stress and proinflammatory responses [84,85].

The impact of isoquinoline alkaloids (IQs) derived from plume poppy (*Macleaya cordata*) extracts on immunological and inflammatory parameters of dogs was studied by [86]. In this study, healthy beagle dogs were fed a commercial supplement containing as active substances the IQs sanguinarine, chelerythrine, allocryptopine and protopine (1.2 g additive/kg feed) during two consecutive three-week periods. No effects were observed on CD3^+^, CD4^+^, CD5^+^, CD8β^+^, CD21^+^ and MHC-II^+^-expressing cells and in serum IgA, IgG and haptoglobin.

The use of a polyherbal choline source was tested by [87]. The authors fed healthy dogs of different breeds with a diet without choline supplementation (containing 377 mg choline naturally derived from the diet/kg of food), a diet supplemented with synthetic choline chloride (3850 mg/kg of a product equivalent to 2000 mg of choline/kg diet) and three inclusion levels of the polyherbal choline source (200, 400 and 800 mg/kg). After comparing the groups fed with the synthetic choline and the 800 mg/kg of the polyherbal, the authors observed a downregulation in cluster of genes associated with inflammation and immune responses, namely the chemokine signaling pathway, cytokine–cytokine interactions and adhesion junction process. This evidence suggests that the polyherbal source may promote anti-inflammatory effects in dogs, which could be beneficial to treat inflammatory pathologies, such as IBD [87].

Overall, the studies available show that different phytonutrients provide a range of health benefits due to their anti-inflammatory, antioxidant and immunomodulatory properties. Carotenoids modulate immune responses, particularly T and B cell functions, promoting an improved in vivo cell-mediated and humoral immunity. In older dogs, supplementation with carotenoids improved T cell responses, counteracting the immune decline associated with aging. Polyphenols modulate gut microbiota and exert anti-inflammatory and antioxidant roles, minimizing the inflammatory status of dogs under different health conditions, such as obesity, stress or osteoarthritis. These findings highlight the potential of phytonutrients as sustainable and functional ingredients for canine nutrition. However, the optimal inclusion level for each ingredient, as well as the duration of treatment and possible side effects, has yet to be established.

**Table 3:** Studies addressing the immune parameters or responses of dogs fed diets containing different phytonutrients.

Phytonutrients				
Breed/Age	Diet	Experimental Design/Duration of the Trial	Immune Parameters	Reference
56 female beagle dogs/17–18 months old	0, 5, 10 or 20 mg of lutein/day (FloraGlo^TM^ crystalline lutein, Kemin Industries Inc., Des Moines, IA, USA) resuspended in soybean oil and administered perorally prior to food	14 dogs per experimental group/12 weeks of feeding/DTH responses measured at weeks 0, 6 and 12; vaccination as antigenic challenge at weeks 13 and 15	↑ DTH responses to PHA and vaccine, lymphocyte proliferative response to ConcA, PHA and PWM, % of CD5^+^, CD4^+^, CD8^+^ and MHC-II^+^ cells and plasma IgG after the second antigenic challenge; No effects on the % of CD21^+^ B cells, IL-2 production by PHA-stimulated PBMCs and plasma IgM	[62]
8 male and 8 female beagle dogs/2 years old	0 and 45 g/kg diet of active xanthophylls (lutein source with 76.7% lutein and 5.2% zeaxanthin)	Completely randomized design with 8 dogs per experimental group/120 days	↑ CD4^+^ and CD8^+^ T cell counts in dogs supplemented with lutein; No effects on lymphocyte proliferation	[63]
56 female beagle dogs/9–10 months old	0, 10, 20 or 40 mg astaxanthin from *Haemotococcus pluvialis*/day	14 dogs per experimental group/16 weeks/vaccination against CDV, CPIV, CAV type-2 and CPV in weeks 12 and 14/intradermal injection with 100 μL of saline, attenuated vaccine and PHA at weeks 12 and 16	Dietary astaxanthin enhanced DTH response to vaccine, ConcA-induced lymphocyte proliferation (with 20 mg at week 12), NK cell cytotoxic activity, plasma IgG and IgM and B cell population; ↓ Plasma CRP in all dogs fed astaxanthin at 16 weeks; No effects on DTH response to PHA, PBMC proliferation response to PHA and PWM and on CD4^+^, CD8^+^ and MHC class II^+^ cell populations	[61]
56 female beagle dogs/4–5 months old	0, 2, 20 or 50 mg β-carotene/day	14 dogs per experimental group/8 weeks	↑ CD4^+^ cell numbers and CD4:CD8 ratio, plasma IgG and DTH responses to vaccine and PHA; ↓ Proliferative response to ConcA and PWM and plasma IgG in low-responder dogs; No effects on IL-2 production by PHA-stimulated PBMCs, plasma IgM and % of CD8^+^, CD5^+^, CD21^+^ and MHC-II^+^ cells	[65]
18 young (9 labrador retrievers and 9 fox terriers) and 18 old (9 labrador retrievers and 9 fox terriers) dogs/mean age 1.7 years old and 10.6 years old, respectively	0, 20 (moderate) or 40 mg/kg diet (high) of β-carotene	9 young and 9 old dogs per experimental group/2 month-periods (moderate β-carotene—period 1; high β-carotene—period 2)	↑ CD4^+^ cell counts and T cell proliferation response to ConcA and PHA in old dogs fed 20 mg/kg of β-carotene compared to old dogs fed the control; ↑ CD4:CD8 ratio, lymphocyte proliferative response to ConcA, PHA and PWM in young dogs fed β-carotene; Old dogs that received 20 mg/kg of β-carotene were not statistically different from young dogs for MHC-II cell distribution; DTH response to PHA of old dogs that received 40 mg/kg of β-carotene not statistically different from young dogs	[66]
56 female beagle dogs/2 years old	0, 5, 10 or 20 mg of bixin/day	14 dogs per experimental group/16 weeks/vaccination with Vanguard 5 at weeks 12 and 14	↑ CD21^+^ B cells and plasma IgG in dogs fed 10 mg; ↑ DTH responses to Vanguard 5 at 12 weeks in dogs fed 20 mg; ↓ Total CD3^+^ cells in dogs fed 10 and 20 mg; ↓ CRP in dogs fed bixin; Variable NK cell activity in dogs fed bixin, depending on the level of bixin and week; No effects on the % of CD4^+^ and CD8^+^ T cells, plasma IgM and IgA, lymphocyte proliferation response to PHA and ConcA and on DTH response to vaccine at week 16	[72]
16 male beagle dogs with induced obesity/13–14 months old	Normal diet (ND; 32.2% fat, 3.885 kcal/day)/high-fat diet (HFD; 53% fat, 5530 kcal/day)/HFD + low level of tea polyphenol (TP; TP 25%; 0.25 g/kg BW)/HFD + high level of TP (TP 50%; 0.50 g/kg BW)	4 dogs per experimental group/12 weeks	↑ COX-2 relative protein levels, and TNF-α, IL-6 and IL-1β mRNA expression in the liver of dogs fed HFD compared to controls; ↓ COX-2 and iNOS relative protein levels and TNF-α, IL-6 and IL-1β mRNA expression in the liver of dogs fed TP diets compared to HFD	[74]
30 male dogs with induced obesity	Normal diet (ND)/high-fat diet (HFD)/HFD + low feeding level of green tea polyphenols (Low GTP; 0.48%)/HFD + medium feeding level of GTP (Medium GTP; 0.96%)/HFD + high feeding level of GTP (High GTP; 1.92%)	6 dogs per experimental group/18 weeks	↑ Serum TNF-α and IL-6, TNF-α and IL-1β mRNA expression in small intestinal epithelial cells and ileal TLR4 protein expression levels in dogs fed HFD compared to controls; ↓ Serum TNF-α, IL-1β and IL-6 and TNF-α, IL-1β and IL-6 mRNA expression in small intestinal epithelial cells and ileal TLR4 protein expression levels in dogs fed GTPs compared to HFD	[75]
15 Chinese indigenous dogs/2 months old	Basal diet (control)/high-fat (HF) diet containing 10% lard/mulberry leaf powder (MLP) diet containing 10% lard and 6% MLP; bioactive compounds in MLP: 1-deoxynojirimycin: 7.06 µg/g, gallic acid: 2.51 µg/g, protocatechuic acid: 9.44 µg/g, gallocatechin gallate: 0.14 µg/g, catechin: 0.04 µg/g, rutin: 657.88 µg/g, quercetin: 3.48 µg/g, chlorogenic acid: 3449.12 µg/g and naringenin: 0.35 µg/g	5 dogs per experimental group/4 weeks	↑ Serum IgA in dogs fed the MLP diet compared to control and HF; No effects of MLP on serum IgG, IgM, IL-6 and TNF-α	[76]
12 healthy and 6 client-owned dogs with diabetes mellitus (DM)	Oral supplementation with turmeric extract capsule (250 mg of curcuminoids per capsule)	6 dogs in the DM group, 12 dogs in the control group/180 days	No effects on plasma IL-6 and IL-10	[77]
12 dogs affected by osteoarthritis (OA) and 6 healthy dogs/4 to 15 years old	0 or 4 mg/kg BW of curcumin, twice a day (CURCUVET^®^, Indena Spa, Milan, Italy; CURCUMIN group)/the OA group receiving oral firocoxib (Previcox^®^, Merial Italia Spa, Milan, Italy) served as positive control (NSAID group)	Randomized controlled trial with 6 dogs per experimental group/20 days	↓ Number of differentially expressed genes involved in “inflammatory response” and “connective tissue development and function” and expression of *TNF*, *TLR4*, *IL8*, *IL18* and *PTGS2* (*COX2*) in both groups, ↑ *IkB* and ↓ *TNF-α*, in the “TNFR1 signaling” pathway, in curcumin group (NSAID did not affect *IkB*); ↓ *TNF-α*, *IL8* and *IL18* in the “role of cytokines in mediating communication between immune cells” in curcumin group (NSAID altered *TNF-α* and *IL8* but not *IL18*)	[78]
74 dogs of different breeds/1–15 years old	Supplementation with 0.10 mg/kg live weight of echinacoside (*Echinacea angustifolia*, EM; Polinacea^®^), 0.20 mg/kg live weight of anthocyanidin (*Vaccinium myrtillus*, VM)*,* 6.60 mg/kg live weight of curcumin (*Curcuma longa*; CL) or 1.5 mg/kg live weight of sylibin (*Sylibum marianum*; SM)	21 healthy dogs in CTRL group, 14 healthy dogs in EA, 13 dogs actively involved in training in VM, 18 dogs with history of arthrosis in CL diet and 8 dogs suffering from liver diseases in SM; all the dogs were fed the CTRL diet 15 days prior the study/60 days	↓ *TNF, NFkB1, CXCL8* and *PTGS2* and ↑ *SOD2* expression in VM and CL; ↓ *TNF* and *NFkB1* and ↑ *CXCL8* expression in EA; ↑ *SOD2* expression in SM; No effects on the expression of *MRPS7*, *PTGS1*, *PPARG*, *IFNG*, *NOS2*, *BAX*, *BCL2L1*, *BCL2*, *NR3C1*, *NR3C2*, *CYP11B2*, *CYP21A2*, *CYP3A4* and *GSTA3*	[80]
18 males and 18 female healthy or with mild inflammatory bowel disease (IBD) labrador retrievers/4.32 ± 1.71 years old	0 (control or vehicle group) or 30 mg/kg BW of grape seed proanthocyanidin (GSP group) dissolved in 10 mL of saline on an empty stomach	12 dogs per experimental group/Experiment 1: 28 days/Experiment 2: dogs from the vehicle group were selected as recipients for fecal microbiota transplantation (FMT); FMT was performed on days 0 and 14, using fresh fecal samples from dogs in the GSP group collected on days 26, 27 and 28 of Experiment 1	↑ Serum CRP, TNF-α, IL-1β and IL-6; ↓ Serum IL-10 in vehicle group, compared to control, after Experiment 1; ↓ Serum CRP, TNF-α, IL-1β and IL-6; ↑ Serum IL-10 in GSP group, compared to vehicle group, after Experiment 1; ↓ Serum CRP, TNF-α, IL-1β and IL-6 in FMT group, compared to vehicle group, after experiment 2; No effects of FMT on serum IL-10	[81]
8 male and 12 female beagle dogs/1.47 ± 0.02 years old	0, 0.02, 0.04 or 0.08% of gallic acid	5 dogs per experimental group/45 days	↓ Serum IL-1β and IFN-γ in dogs fed gallic acid; No significant effects on serum TNF-α, IL-8 and IgG	[83]
8 male and 11 female beagle puppies/3.5 months old	0 or 500 mg/kg of gallic acid (GA) and transportation stress (TS)	6 dogs in the control group, 6 dogs in TS group and 7 dogs in the TS + GA group/2 weeks	↑ Serum IgG and IL-4 in TS-GA after transportation compared to TS; No significant differences in TNF-α and IFN-γ in TS + GA after transportation compared to controls	[84]
13 beagle puppies/3.56 ± 0.24 months	2.5 g/kg gallnut tannic acid (TA) and induced stress (ST)	6 dogs in the ST and 7 dogs in the ST + TA group/2 weeks/dogs were maintained in a room at 29 ± 1 °C and a relative humidity of 96 ± 3%, from day 1 to day 7; transportation for 3h to an environment under a temperature of 23% and humidity of 70% from 8 to 14 days	Tendency for a higher serum IL-2 (*p* < 0.10) in ST + TA on day 1 before transportation compared to ST; Tendency for a higher serum IL-4 and lower serum IL-6 (*p* < 0.10) ST + TA group after transportation compared to ST; No effects of TA on serum TNF-α	[85]
2 male, 4 castrated male and 4 female beagle dogs/1–5 years old	0 or 1.2 g/kg feed of Sangrovit^®^ Extra (granulated and standardized extract of *Macleaya cordata*; Phytobiotics, Eltville, Germany); the additive provides the isoquinoline alkaloids (IQs) sanguinarine, chelerythrine, allocryptopine and protopine as active substances	Cross-over design with two consecutive three-week periods	No effects on CD3^+^, CD4^+^, CD5^+^, CD8β^+^, CD21^+^ and MHC-II^+^ cells and serum IgA, IgG and haptoglobin	[86]
20 male and 20 female dogs of different breeds/4.6 ± 1.6 years old	Unsupplemented diet (377 mg choline naturally derived from the diet/kg of food); choline chloride (3850 mg/kg of a synthetic product equivalent to 2000 mg of choline/kg of food); 200, 400 and 800 mg/kg of a poyherbal choline (BioCholine)	Completely random design with 8 animals per experimental group/60 days/transcriptome analysis performed in dogs fed with 2000 mg synthetic choline and 800 mg polyherbal	↓ Genes associated with chemokine signaling pathway, adhesion junction process and cytokine–cytokine receptor interaction	[87]

BAX: Apoptosis regulator BAX gene; BCL2: Apoptosis regulator gene; BCL2L1: BCL2-like 1 gene; CAV type-2: Canine adenovirus type-2; CD: Cluster of differentiation; CDV: Canine distemper virus; ConcA: Concanavalin A; COX2: Cyclooxygenase-2 (PTGS2); CPIV: Canine parainfluenza virus; CPV: Canine parvovirus; CRP: C-reactive protein; CXCL8: C-X-C motif chemokine ligand 8 gene (IL-8); CYP11B2: Cytochrome P450 family 11 subfamily B member 2 gene; CYP21A2: Cytochrome P450 family 21 subfamily A member 2 gene; CYP3A4: Cytochrome P450, family 3, subfamily A, polypeptide 4 gene; DTH: Delayed-type hypersensitivity response; GSTA3: Glutathione S-transferase alpha 3 gene; IFNG: Interferon-gamma gene; IFN-γ: Interferon-gamma; Ig: Immunoglobulin; IkB: Inhibitor of nuclear factor kappa B gene; IL: Interleukin; IL8: Interleukin 8 gene; IL18: Interleukin 18 gene; iNOS: Inducible nitric oxide synthase; MHC-II: Major histocompatibility complex class II; MRPS7: Mitochondrial ribosomal protein S7 gene; NFkB1: Nuclear factor kappa B subunit 1 gene; NOS2: Nitric oxide synthase 2 gene; NK cell: Natural killer cell; NR3C1 and 2: Nuclear receptor subfamily 3 group C member 1 and 2 genes; NSAID: Non-steroidal anti-inflammatory drug; PBMCs: Peripheral mononuclear cells; PHA: Phytohemagglutinin; PPARG: Peroxisome proliferator-activated receptor gamma gene; PTGS1 and 2: Prostaglandin-endoperoxide synthase 1 and 2 genes; PWM: Pokeweed mitogen; SOD2: Superoxide dismutase 2 gene; TNF: Tumor necrosis factor gene; TNF-α: Tumor necrosis factor-alpha; TNFR1: Tumor necrosis factor receptor 1; TLR4: Toll-like receptor. Upward arrows (↑) indicate an increase, while downward arrows (↓) indicate a decrease in the parameters analyzed.

## 4. Conclusions and Future Directions

**Figure 4:**
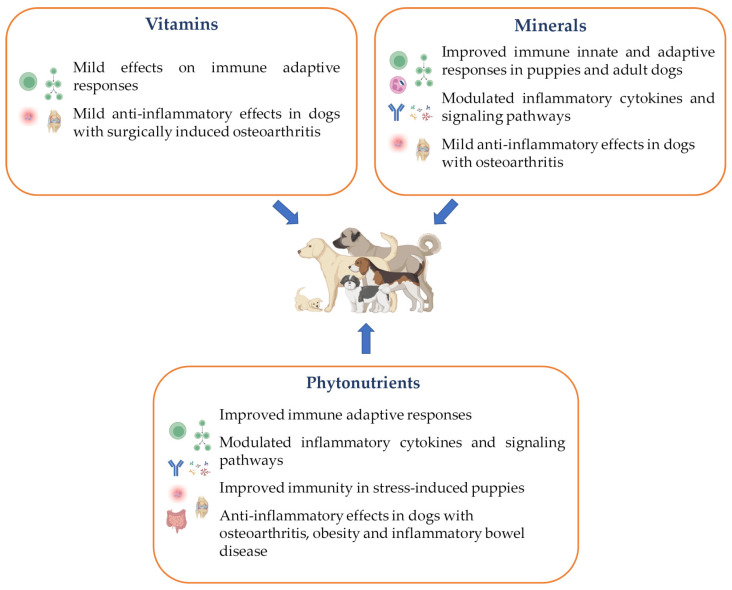
Main effects of dietary vitamins, minerals and phytonutrients on canine immune and inflammatory responses.

The phenomenon of pet humanization has resulted in increased concern regarding the health and wellness of companion animals, with owners demonstrating a willingness to invest in diets with functional properties. However, while many commercial diets claim to support health and immune responses in pets, scientific evidence supporting these claims remains limited. This literature review summarizes the current knowledge on the immunomodulatory properties of vitamins, minerals and phytonutrients in dogs (Figure 4), identifying research gaps that must be addressed to develop tailor-made diets that meet the unique needs of canines, depending on their age and health condition. Although the use of these functional ingredients holds promise for supporting effective immune responses in dogs, the limited number of studies available does not yield robust conclusions on their benefits due to variations in breed, age and physiological conditions among subjects. Many of these studies were conducted in healthy young and adult canines, for a short period of time, and the long-term effects of these compounds are still unknown. Moreover, the functional ingredients that were tested are quite variable, as well as the dietary levels administered. Consequently, the studies discussed in the present review should be interpreted with caution, as variations in ingredients, inclusion levels, administration periods, nature of the basal diet and dogs’ breed, age and lifestyle might significantly influence the observed outcomes.

Depending on the breed, age or lifestyle, dogs can suffer from a variety of inflammatory diseases, including osteoarthritis, IBD and obesity [12]. Some of these studies investigated the effects of vitamins, minerals and phytonutrients in animal models with various natural or induced pathological conditions, demonstrating potential benefits in mitigating inflammation associated with these diseases. Yet, further research is necessary to elucidate how functional supplements influence the immune responses, both at the cellular and molecular levels, and to determine the appropriate feeding levels and optimal duration of administration. Such studies are essential to understand the mechanisms behind the anti-inflammatory and immunomodulatory properties of these compounds and will aid in the development of therapeutic approaches based on dietary supplementation that can complement conventional treatments.

It is well-known that dogs experience changes in the immune system across distinct stages of life. With advancing age, a decline in their immune responses is observed, which can increase their susceptibility to infections and inflammatory or chronic diseases [88]. Studies in senior dogs indicate an impairment of cell-mediated immunity, with reduced blood CD4^+^ T cells and a lower response of blood lymphocytes to stimulation by mitogens. Conversely, puppies are at risk of developing infectious diseases as their immune system is still undergoing maturation [88]. While studies conducted in puppies and aged dogs suggest an improvement of immune status with dietary supplementation of different functional additives (organic selenium, curcumin, gallic and gallnut tannic acids and β-carotene), there is a significant lack of evidence regarding the potential influence of these compounds in the immune system of these dogs. Thus, testing functional supplements specifically in these groups comprises a future research area that needs to be addressed, due to their unique immune profile and health challenges. Moreover, the considerable variability in domestic dogs is also critically important to consider for commercial applications and to support potential claims.

## Data Availability

No new data were created or analyzed in this study. Data sharing is not applicable to this article.
